# P-1495. Antibiotic and Phage Susceptibility of Pseudomonas aeruginosa Clinical Isolates From Phage Therapy Patients

**DOI:** 10.1093/ofid/ofae631.1665

**Published:** 2025-01-29

**Authors:** Justin J Lee, Sergei Elber Dorozko, Ryan K Shields, Ghady Haidar, Daria Van Tyne

**Affiliations:** University of Pittsburgh School of Medicine, San Diego, California; University of Pittsburgh, Pittsburgh, Pennsylvania; University of Pittsburgh, Pittsburgh, Pennsylvania; University of Pittsburgh School of Medicine, San Diego, California; University of Pittsburgh School of Medicine, San Diego, California

## Abstract

**Background:**

Antibiotic resistance is a rising issue, with the World Health Organization predicting that by 2025, around 10 million deaths annually will be caused by resistant infections. Phage therapy is an alternative form of treatment for multidrug-resistant bacterial infections in which bacterial viruses, called bacteriophages, are used to combat the infection. Before phage therapy can be delivered at the bedside, clinical bacterial isolates from the patient are collected and screened for susceptibility in the laboratory to identify candidate phages that can infect and kill the bacteria causing the infection. Many phages have been isolated from wastewater and assembled into libraries without detailed knowledge of their host range or an understanding of which phages are the best performers.

**Methods:**

Using 40 patient-derived *P. aeruginosa* clinical isolates collected from 21 phage therapy candidate patients at the University of Pittsburgh Medical Center in the years 2022 and 2023, we investigated the host range of 25 phages in a *P. aeruginosa* screening library. We also screened the isolates against 11 antibiotics currently used to treat *P. aeruginosa* infections.

**Results:**

We identified correlations in phage susceptibility profiles both within and between patients’ isolates, finding that a three phage cocktail could target 100% of the isolates. We also examined the antimicrobial susceptibility profiles of the 40 isolates and found that 67.5% of isolates are multidrug resistant.

**Conclusion:**

With this research, we hope to develop methods for identifying candidate phages more quickly, speeding up the process of phage susceptibility testing, and aiding in the assembly of highly active phage cocktails, which may result in increased accessibility of phage therapy to a more diverse patient base.

**Disclosures:**

**Ryan K. Shields, PharmD, MS**, Allergan: Advisor/Consultant|Cidara: Advisor/Consultant|Entasis: Advisor/Consultant|GSK: Advisor/Consultant|Melinta: Advisor/Consultant|Melinta: Grant/Research Support|Menarini: Advisor/Consultant|Merck: Advisor/Consultant|Merck: Grant/Research Support|Pfizer: Advisor/Consultant|Roche: Grant/Research Support|Shionogi: Advisor/Consultant|Shionogi: Grant/Research Support|Utility: Advisor/Consultant|Venatorx: Advisor/Consultant|Venatorx: Grant/Research Support **Ghady Haidar, MD**, Allovir: Grant/Research Support|AstraZeneca: Advisor/Consultant|AstraZeneca: Honoraria|Cystic Fibrosis Foundation: Grant/Research Support|Karius: Advisor/Consultant|Karius: Grant/Research Support|NIH: Grant/Research Support|PeerView Institute for Medical Education: Honoraria|PRIME: Honoraria|Regeneron: Grant/Research Support

